# The First Genome Survey of the Snail *Provanna glabra* Inhabiting Deep-Sea Hydrothermal Vents

**DOI:** 10.3390/ani13213313

**Published:** 2023-10-25

**Authors:** Min Hui, Yu Zhang, Aiyang Wang, Zhongli Sha

**Affiliations:** 1 Department of Marine Organism Taxonomy & Phylogeny, Institute of Oceanology, Chinese Academy of Sciences, Qingdao 266071, China; minhui@qdio.ac.cn (M.H.); wangaiyang@qdio.ac.cn (A.W.); 2 Laoshan Laboratory, Qingdao 266237, China; 3 Shandong Province Key Laboratory of Experimental Marine Biology, Institute of Oceanology, Chinese Academy of Sciences, Qingdao 266071, China; 4 College of Marine Life Sciences, Ocean University of China, Qingdao 266100, China; zy1544@stu.ouc.edu.cn

**Keywords:** deep-sea chemosynthetic ecosystem, genome size, molecular marker, transposal elements, phylogenetics

## Abstract

**Simple Summary:**

Due to limited reporting of genomic resources for deep-sea organisms, studies of their adaptive mechanisms for extreme environments have been hindered. A genome survey of the snail *Provanna glabra*, a representative species in deep-sea chemosynthetic ecosystem, was conducted based on Illumina sequencing. Genome size, repetitive elements, heterozygosity, and other genome features were estimated and characterized. A draft genome and a complete mitochondrial genome were assembled for *P. glabra*, and candidate molecular markers were identified. Phylogenetic analysis based on mitochondrial genes was performed. The results provide effective genome information for *P. glabra* and supply a basis for further high-quality genome map construction and biogeography study of this deep-sea chemosynthetic snail.

**Abstract:**

The snail *P. glabra* is an endemic species in deep-sea chemosynthetic ecosystems of the Northwest Pacific Ocean. To obtain more genetic information on this species and provide the basis for subsequent whole-genome map construction, a genome survey was performed on this snail from the hydrothermal vent of Okinawa Trough. The genomic size of *P. glabra* was estimated to be 1.44 Gb, with a heterozygosity of 1.91% and a repeated sequence content of 69.80%. Based on the sequencing data, a draft genome of 1.32 Gb was assembled. Transposal elements (TEs) accounted for 40.17% of the entire genome, with DNA transposons taking the highest proportion. It was found that most TEs were inserted in the genome recently. In the simple sequence repeats, the dinucleotide motif was the most enriched microsatellite type, accounting for 53% of microsatellites. A complete mitochondrial genome of *P. glabra* with a total length of 16,268 bp was assembled from the sequencing data. After comparison with the published mitochondrial genome of *Provanna* sp. from a methane seep, 331 potential single nucleotide polymorphism (SNP) sites were identified in protein-coding genes (PCGs). Except for the *cox1* gene, *nad2*, *nad4*, *nad5*, and *cob* genes are expected to be candidate markers for population genetic and phylogenetic studies of *P. glabra* and other deep-sea snails. Compared with shallow-water species, three mitochondrial genes of deep-sea gastropods exhibited a higher evolutionary rate, indicating strong selection operating on mitochondria of deep-sea species. This study provides insights into the genome characteristics of *P. glabra* and supplies genomic resources for further studies on the adaptive evolution of the snail in extreme deep-sea chemosynthetic environments.

## 1. Introduction

Deep-sea hydrothermal vent is one of the chemosynthetic ecosystems primarily relying on chemoautotrophs to sustain their productivity and is characterized by high hydrostatic pressure, low oxygen, and being rich in hazardous substances, such as heavy metals, methane, and hydrogen sulfide [1,2]. Vents are usually found in active mid-ocean ridges and back-arc diffusion centers, and during the eruption, the fluid’s temperature can exceed 400 °C [3,4]. Despite the harsh environment, many macrobenthos are found densely distributed around hydrothermal vents, e.g., fish, polychaetes, crustaceans, and mollusks. However, how these organisms adapt to the extreme environment remains unclarified.

Genome information could provide insights into the evolution and adaptive mechanisms of lives inhabiting deep-sea chemosynthetic ecosystems [5,6,7]. Benefiting from the development of next-generation sequencing technologies, whole-genome maps of several animal species from hydrothermal vents or cold seeps have been completed, and the genetic mechanisms of their adaptation to the extreme environments have been explored in bivalve, gastropod, tubeworm, and sea cucumber [8,9,10,11]. However, genome resources for deep-sea vent and seep species are still scarce in comparison with shallow-water species. Before whole-genome sequencing and assembly, it is necessary to study the genome size and characteristics of the target species in order to decide the sequencing strategy and depth.

Gastropod is a dominant group in deep-sea chemosynthetic ecosystems [12]. The Provannidae family of gastropods is large in number and widely distributed in hydrothermal vents, cold seeps, sunken wood, and vertebrate falls [12,13,14]. *Provanna glabra* Okutani, Tsuchida & Fujikura, 1992 belonging to Provannidae is one of the common species endemic to the deep-sea hydrothermal vent and cold seep areas in the Northwest Pacific. Thus far, only the mitochondrial genome of *Provanna* sp. is assembled, and phylogenetic analyses are conducted for *Provanna* snails and other related species [15]. Therefore, more genetic and genomic resources for this typical gastropod in chemosynthetic ecosystems are very important and imperative to facilitate the study of its adaptive evolution to extreme environments.

Genome survey sequencing (GSS) is an efficient approach for generating genome information, which can serve as a reference for population genomic and evolution studies and supply background for the subsequent whole-genome map construction. In this study, GSS for *P. glabra* from a hydrothermal vent in the Okinawa Trough was performed, and sequences were assembled into a draft genome. Genome size, heterozygosity, and repeat content were estimated, and candidate molecular markers were identified. A mitochondrial genome was also assembled, and phylogenetic analysis for gastropods was conducted. Candidate molecular markers were also identified. The results are expected to add more genomic resources and provide a basic understanding of the *P. glabra* genome. 

## 2. Materials and Methods

### 2.1. Sample Collection and DNA Extraction

In this study, only one snail from the deep sea was used for the experiment, and all efforts were made to minimize the suffering of the snail. The experiments were conducted in strict accordance with the guidelines of the Regulations on the Management of Animal Experiments (20170301, https://www.gov.cn/gongbao/content/2017/content_5219148.htm, accessed on 10 July 2018) constituted by the Chinese government, and the Regulations on the Management of Animal Experiment Safety (2020-37) constituted by the Institute of Oceanology, Chinese Academy of Sciences.

**Figure 1:**
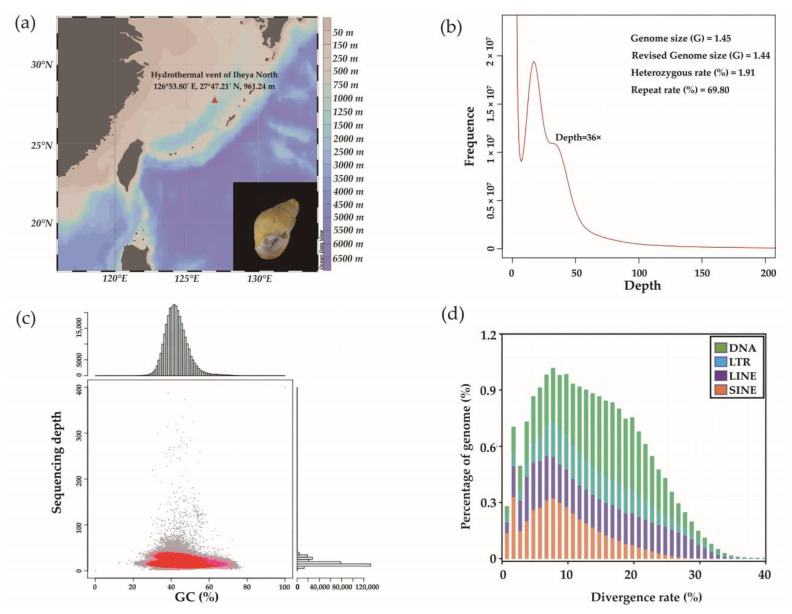
Sampling site and genome characteristics of *P. glabra*. (**a**) Map showing the sampling area of *P. glabra.* It is drawn by Ocean Data View (ODV) v.5.0. (**b**) *K*-mer (*K* = 17) analysis for the genome. (**c**) Correlation between GC content and sequencing depth of the assembled draft genome. The horizontal axis represents GC content, and the vertical axis represents sequencing depth. The red portion represents the denser area of points in this scatterplot. The rightmost plot shows the distribution of sequencing depth of contigs, with the horizontal coordinate representing the number of contigs. The topmost plot displays the distribution of GC content, with its vertical coordinate indicating the number of contigs. (**d**) Divergence rates of four TE types in *P. glabra* draft genome.

In July 2018, samples of *P. glabras* were collected from the Iheya North hydrothermal vent in Okinawa Trough (126°53.80′ E, 27°47.21′ N, depth 961.24 m) (Figure 1a) during the cruise by the scientific research vessel (RV) KEXUE (Institute of Oceanology, Chinese Academy of Sciences, China). Samples were taken by the remotely operated vehicle (ROV) Quasar MkII on RV KEXUE, frozen in liquid nitrogen, and stored at −80 °C for subsequent DNA extraction. 

### 2.2. DNA Extraction and Sequencing 

DNA of the muscle tissue of the individual was extracted with the traditional phenol-chloroform method [16], and its quality and concentration were assessed by 1.0% agarose and a NanoDrop 2000 spectrophotometer. The tested DNA was used for sequencing library construction using the Illumina library preparation kit (Illumina, San Diego, CA, USA) according to the instructions. The paired-end libraries with an insert length of 350 bp were then sequenced on the Illumina Hiseq X-ten sequencing system (Illumina, San Diego, CA, USA).

### 2.3. Quality Control, K-mer Analysis, and Assembly of Sequences

The raw reads were then filtered using Trimmomatic v.0.39 [17] to produce clean reads for further analysis. Data filtering was strictly performed according to the following criteria: (i) trimming reads with adaptors; (ii) deleting low-quality reads with unidentified nucleotides (N) content more than 8%; (iii) removing reads with a high percentage (>20%) of low-quality bases (quality score less than 10). *K*-mer (*K* = 17) analysis was performed by Jellyfish to calculate genome size, repetitive sequence content, and heterozygosity [18]. The draft genome was de novo assembled based on high-quality reads with SOAPdenovo 2 [19] by setting *K*-mer = 41. Completeness of the assembled genome was estimated against the metazoa_odb10 database using BUSCO (Benchmarking Universal Single-Copy Orthologs) v.5.5.0 [20].

### 2.4. Annotation of Repetitive Sequences and Identification of Microsatellites

Repeat sequence identification was performed on the assembled genome. Tandem repeats in the genome were identified through TRF (Tandem Repeats Finder) 4.0 (http://tandem.bu.edu/trf/trf.html, accessed on 26 December 2022). For interspersed repeats (TEs) in the genome, a repeat sequence database of the genome was set up by de novo prediction of RepeatModeler (http://www.repeatmasker.org/RepeatModeler/, accessed on 28 December 2022) and LCR-FINDER [21], and then RepeatMasker was further used to annotate and classify the repeat sequences. Meanwhile, RepeatMasker (http://www.repeatmasker.org/, accessed on 28 December 2022) and RepeatProteinMask were selected to identify TEs by comparing genome sequences with the reported repeat sequences in the RepBase database [22]. The divergence rates of different types of TEs were calculated by using RepeatMasker based on the merged repeat sequence database. Simple sequence repeats (SSRs) in the genome were detected using the MIcroSAtellite identification system (MISA) v. 2.1 [23].

### 2.5. Mitochondrial Genome Assembly and SNP Screening

With clean reads, the mitochondrial genome of *P. glabra* was assembled by NOVOPlasty v. 4.2.1 [24]. During assembly, the mitochondrial cytochrome *c* oxidase subunit I (*cox1*) sequence of *P. glabra* was used as a seed file and *K*-mer was set as 33. The newly assembled mitochondrial genome was annotated on the MITOS2 website (http://mitos2.bioinf.uni-leipzig.de/, accessed on 18 April 2023) and Geseq (https://chlorobox.mpimp-golm.mpg.de/geseq.html, accessed on 18 April 2023) followed by manual correction. The reassembled mitochondrial genome sequence was compared with the previously published mitochondrial genome sequence of *Provanna* sp. [15] using BioEdit version 7.2 in order to identify candidate SNPs. Nucleotide sequences of PCGs were translated into amino acid sequences on the NovoPro website (https://www.novopro.cn/tools/translate.html, accessed on 20 April 2023). The non-synonymous loci were then screened by comparing the amino acid sequences of the two mitochondrial genomes with BioEdit version 7.2.

### 2.6. Phylogenetic and Selective Pressure Analyses of Mitochondrial Genes

From the NCBI database, we downloaded complete mitochondrial genome sequences of another 20 gastropod species, including four other species from a deep-sea vent or seep environments and one cephalopod species (*Amphioctopus marginatus*, outgroup) (Table S1). Based on the TBtools-II bioinformatics platform [25], 13 PCG sequences of 22 species were compared using MUSCLE, and a phylogenetic tree was constructed using IQ-tree under an mtInv+F+R4 model of nucleotide sequences with maximum likelihood (ML) method. By setting the clade of deep-sea chemosynthetic species as foreground lineage (Abyssochrysodea) and shallow-water species, *Bolinus brandaris*, *Conus borgesi*, and *Monoplex parthenopeus* as background lineage, EasyCodeML was used to evaluate the selection pressure in the mitochondrial genes from different lineage [26]. With the branch model, the ω (dN/dS) value of the one-ratio Model 0, the ω0 of the background lineage, and the ω1 of the foreground lineage of the two-ratio Model 2 were evaluated, respectively. The two models were compared according to the likelihood ratio test (LRT, *p*-value < 0.05) to determine whether the evolutionary rate (dN/dS) of deep-sea species was different from that of shallow-water species.

## 3. Results and Discussion

### 3.1. Genome Sequencing, Size Estimation and Assembly

Genomic sequencing of *P. glabra* generated a total of 63.54 Gb raw reads. After filtering, a total of 55.70 Gb of clean data was obtained with Q30 at 92.98% and GC content at 44.48%, indicating high sequencing quality (Table 1). The clean data were deposited in the NCBI SRA database with the accession number PRJNA990319. By *K*-mer analysis, the estimated genome size was 1.45 Gb. After correction, genome size was revised into 1.44 Gb with heterozygous rate and repetitive sequence rate of 1.91% and 69.80%, respectively (Figure 1b), indicating a complex genome. Compared with other snails from deep-sea chemosynthetic environments, such as *Ifremeria nautilei* (0.88 Gb), *Alviniconcha hessleri* (0.92 Gb), *Chrysomallon squamiferum* (~0.46 Gb), and *Gigantopelta aegis* (~1.29 Gb), *P. glabra* exhibited relative larger genome sizes, as well as high heterozygosity and repeat content [27,28]. Considering the function of repetitive elements in genome evolution [29], the accumulation of repetitive sequences might contribute to the genome size variation among *P. glabra* and other deep-sea snails. 

Genome assembly resulted in 4,132,680 contigs with an N50 length of 437 bp and 3,534,913 scaffolds with an N50 length of 581 bp (Table 1). The total length of the scaffolds was 1.32 Gb, covering about 92% of the genome, and the GC content of the assembly was 45.52%. GC statistics on the assembly showed that the GC content of sliding windows was populated without other independent sequence clusters, indicating that the sequencing sample was not contaminated by alien species (Figure 1c). BUSCO analysis showed that 67.19% of orthologs were missing in the assembled draft genome of *P. glabra* (Table S2). Therefore, it shows that the quality of the genome assembly is low only based on Illumina short-read sequencing data. Long-read sequencing technologies, such as PacBio SMRT (Single Molecule Real Time) and ONT (Oxford Nanopore Technology, Oxford, UK), are recommended in the subsequent high-quality genome map construction of *P. glabra*. Moreover, Hi-C (High-throughput chromosome conformation capture) technology can be used to link contigs to different chromosomes. In this study, the genome survey results have provided preliminary insights into genome characteristics of *P. glabra*, laying the foundation for further whole-genome sequencing and assembly and significantly enriching genomic resources of deep-sea life.

### 3.2. Repeated Elements in the Genome of P. glabra

Repeat elements, especially transposon, are an important part of the eukaryotic genome and a source of genetic variation [30,31,32]. In total, 636 Mb of repeated elements were screened out, representing 48.03% of the entire assembled draft genome of *P. glabra* (Table S2), in which, 532 Mb of TEs were included. DNA transposon (9.39%) was the most dominant type among all TEs. It was followed by long interspersed elements (LINEs, 6.17%), short interspersed elements (SINEs, 4.53%), and long terminal repeats (LTRs, 3.75%) (Table 2). Comparing the TE content of *P. glabra* with that in seven other gastropod species with genome assembly (three from deep sea and four from the shallow sea (Table S4) [28,33,34,35], it has been found that DNA transposons are the most dominant type in deep-sea gastropods, while in most shallow-water species, LINEs are abundant. It indicates that DNA transposons might play important roles in the genome evolution of deep-sea gastropods.

Observing the divergence rate of TEs can provide us with a deeper understanding of the evolutionary history of TEs. A higher divergence rate represents a greater number of mutations accumulated, which indicates that TEs have existed in the genome for a longer period, while conversely, it suggests a shorter insertion time. Most TEs in the *P. glabra* genome were found with peaks of the divergence rate lower than 10% except that the peak of divergence rate in DNA transposon was lower than 20% (Figure 1d). It indicates that most TEs in the genome of *P. glabra* burst recently and are relatively young [36]. Moreover, it has been reported that TEs can produce a variety of mutations leading to high genomic plasticity and are sensitive to environmental changes [37,38,39]. For example, a substantial proportion of TE insertions and their frequency in *Drosophila* are correlated with environmental variables, such as temperature and rainfall [40]. Besides functioning in local adaptation, TEs in *Drosophila* have also been involved in resistance to microbial infection and toxic substances by inserting themselves into protein-coding sequences of key genes [41]. More importantly, in another study of population genetics of *P. glabra* from different chemosynthetic environments, some TEs were discovered to be under selection and involved in the adaptation to the local environment [42]. Hence, these findings together with our results suggest that TEs within *P. glabra* might be rapidly activated during its invasion of deep-sea chemosynthetic environments, and the proliferation of young TEs in *P. glabra* might function in the adaptive evolution of this snail.

### 3.3. Microsatellites in the Genome of P. glabra

**Figure 2:**
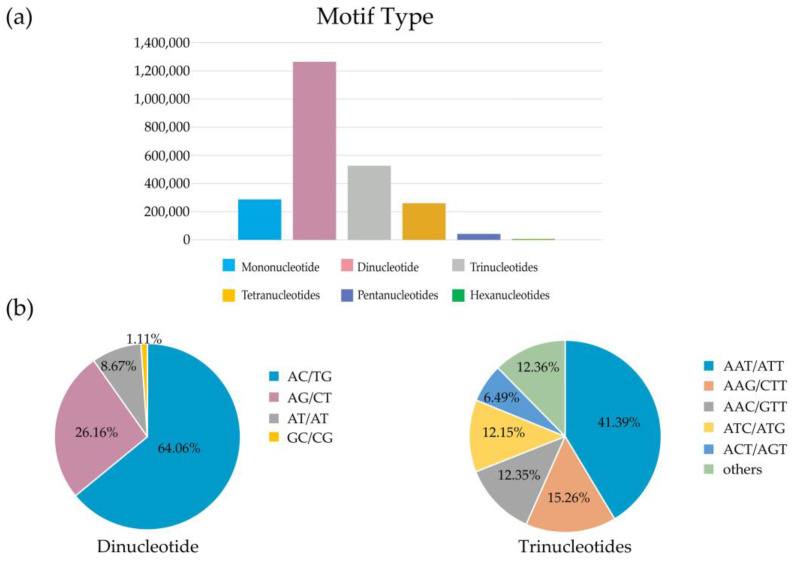
Statistics on microsatellite motifs in *P. glabra* draft genome. (**a**) Distribution of different microsatellite motif types. (**b**) Frequency of different dinucleotide and trinucleotide microsatellite motifs.

Microsatellites, also known as SSRs, are widely distributed in the genomes of both eukaryotes and prokaryotes, usually in the form of low repeats of 1–6 bp. A total of 2,387,170 microsatellite loci were found in the *P. glabra* genome assembly. Statistics of microsatellite loci showed that the majority of them were dinucleotide motifs (53%), followed by trinucleotide (22.04%), tetranucleotide (10.89%), pentanucleotide (1.77%), and hexanucleotide motifs (0.29%) (Figure 2, Table S5). The most prevalent patterns in each motif type were AC/GT (64.06%) in dinucleotides, AAT/ATT (41.39%) in trinucleotides, AAAC/GTTT (23.63%) in tetranucleotides, AATAC/ATTGT (20.78%) in pentanucleotides, and ACAGAG/CTCTGT (17.47%) in hexanucleotides. With characteristics of high polymorphism and codominant, microsatellites are excellent markers in genetic diversity and genetic structure studies [43], and polymorphism is usually higher in dinucleotide and trinucleotide motifs than in other microsatellite types. Therefore, in the future study of the biogeography of *P. glabra*, dinucleotide, and trinucleotide SSRs should be prioritized as molecular markers [44]. Our study supplies the basis for marker development of the deep-sea snail. Moreover, once the high-quality genome map of *P. glabra* is completed, population re-sequencing based on the genome is expected to provide more insights into the genetic evolution of the species.

### 3.4. Phylogeny Based on Mitochondrial Genomes and SNP Candidates

**Figure 3:**
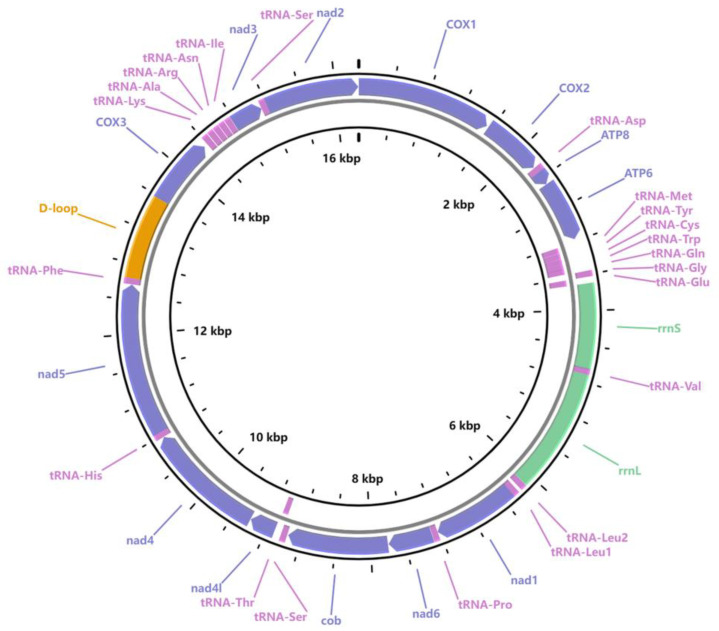
Mitochondrial genome map of *P. glabra*. Purple, pink, and green represent PCGs, tRNA genes, and rRNA, respectively. The control region is marked in yellow.

Mitochondrial genes are frequently employed in phylogenetic and population genetic analyses. A complete mitochondrial genome of *P. glabra* with a length of 16,268 bp was assembled from the genome survey data and deposited in GenBank under the accession number OR209184 (Figure 3). After annotation, we identified 13 PCGs, 2 rRNA genes, and 22 tRNA genes (Table S6).

The obtained mitochondrial genome sequence shows high similarity to that of *Provanna* sp. Published by Xu et al., 2015 [15], in which the snail was collected from a methane seep on the continental slope of the South China Sea. A longer control region (934 bp VS. 856 bp) was obtained in our study. By sequence alignment of 13 PCGs in the two mitochondrial genomes, a total of 331 potential SNPs were identified (Table 3). Among the PCGs, the *cox3* gene exhibited the highest mutation rate (3.72%), followed by *cob* (3.68%), while the *atp6* and *atp8* genes had the lowest proportion of mutations. It was also noted that most *nad* genes showed a high proportion of mutations (2.02~3.67%) (Table 3). *Cox1* is usually used as a molecular marker to distinguish inside and interspecific genetic evolutionary information [45,46]. In this study, *cox1* with a length of 1536 showed a mutation rate of 2.15%. Considering the high mutation rate (>3%) and sequence length (>1000 bp, proper for primer design) of genes, *nad2*, *nad4*, *nad5*, and *cob* are suggested to be candidate markers utilized for phylogenetic and population genetic analysis of *P. glabra* and other deep-sea snails.

Mitochondria in eukaryotes can provide energy for cell life by driving oxidative phosphorylation through the respiratory chain and proton pump, and it has been demonstrated that mitochondrial genes would be under selection in extreme environments [47]. Therefore, we performed phylogenetic analysis and examined selection pressure on mitochondrial genes in deep-sea gastropods, including *P. glabra*.

**Figure 4:**
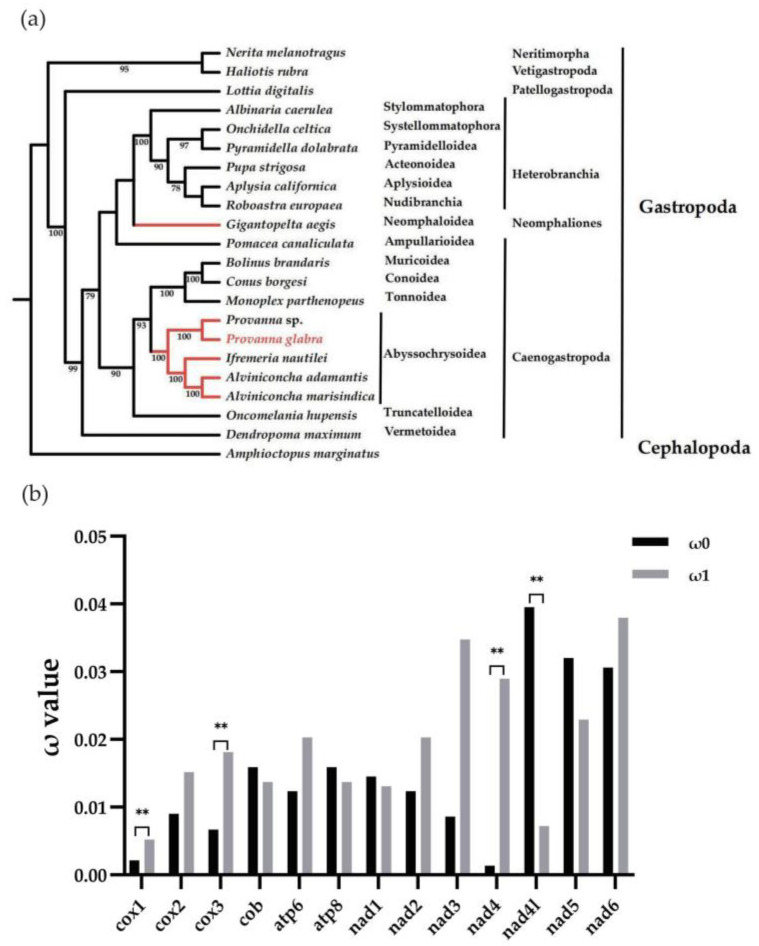
Phylogenetic tree and evolutionary rates of gastropod mitochondrial genes. (**a**) Phylogenetic tree of gastropods with *Amphioctopus marginatus* as outgroup. Bootstrap value is displayed on the branch. Red branches represent deep-sea gastropods. (**b**) Evolutionary rate comparison of mitochondrial genes between deep-sea lineage (Abyssochrysoidea) and shallow-water lineage (Tonnoidea, Conoidea, and Muricoidea). ω0 represents the dN/dS values of the shallow-water lineage, and ω1 represents the dN/dS values of the deep-sea lineage. ** indicates extremely significant difference with 0.001 < *p*-value < 0.01.

Based on the 13 PCGs, a phylogenetic tree of gastropoda was constructed (Figure 4a). It was found that *P. glabra* and four other species from deep-sea vents or seeps clustered together and all belonged to Abyssochrysoidea. *Provanna glabra* showed the closest relationship with *Provanna* sp. By further comparison of mitochondrial PCGs of *Provanna* sp and *P. glabra* from different seep and vent environments, 20 non-synonymous sites were identified, most of which were located in *nad* genes (Table 3). Specifically, six and five non-synonymous sites were detected in *nad2* and *nad5*, followed by *nad3* with three non-synonymous sites. One non-synonymous site was found in *nad4*, *nad4l*, and *nad6*, respectively. Meanwhile, the non-synonymous mutation was also detected in the *cox3* (two sites) and *cob* (one site) gene. It indicates that the mutational events occurring in these mitochondrial genes may impact the adaptive evolution process of *Provanna* snails from different vent and seep environments [48,49,50,51,52]. In other words, the isolation and environmental differences of vent and seep might promote the genetic divergence of the two related species as suggested in the study for vent and seep squat lobsters and mussels [53,54].

Moreover, a positive selection of mitochondrial genes has been also observed in various deep-sea benthic animals, such as mussels, shrimps and crabs [46,55,56]. In our study, the selection pressure suffered in different lineages was also estimated. Based on the phylogenetic tree, ω (dN/dS) values of deep-sea Abyssochrysoidea lineage (foreground branch) and shallow-water lineage (Tonnoidea, Conoidea, and Muricoidea, foreground branch) were calculated and compared. The results based on the branch model showed that in both M0 and M2 models, ω (dN/dS) values for all PCGs were lower than 1, signifying strong purifying selection operating on the mitochondrial genomes within the two gastropod lineages (Table S7). When compared, ω values of four genes were significantly different (*p* < 0.01) between the two lineages, suggesting different evolutionary rates (Figure 4b, Table S7). Among them, *cox2*, *cox3*, and *nad4* exhibited higher ω values in deep-sea species compared with the shallow-water species, with notable distinctions observed in the *nad4* gene. It is known that NADH dehydrogenase is the largest and most crucial proton pump in the respiratory chain [49,52]. The significant evolutionary rate differences in the *nad* genes between deep-sea and shallow-water gastropods suggest that such genes may experience greater selective pressure in the adaptive evolution of deep-sea gastropods. Additionally, cytochrome oxidase has been demonstrated to play an important role in anaerobic adaptation, with its catalytic core encoded by three PCGs (*cox1*, *cox2*, *cox3*) [57]. Therefore, the rapid evolution of *cox2*, and *cox3* in deep-sea gastropods might aid them in better adapting to deep-sea anaerobic environments and other stressors. In summary, our results reveal that the mitochondrial *cox* and *nad genes* in deep-sea gastropods not only are under selection in different vent and seep environments but have also evolved at a distinct rate when compared with shallow-water species, indicating their important roles in the adaptive evolution of deep-sea gastropods.

## 4. Conclusions

In this study, a genome survey of *P. glabra* from a hydrothermal vent in Okinawa Trough revealed a complex snail genome, with a relatively large genome size, high heterozygosity, and repeat sequence. Sequence assembling resulted in a draft genome of *P. glabra*. In the genome assembly, TEs were found inserted in the genome recently and they accounted for high proportions of the genome, which might contribute to the genome evolution of the snail. Large numbers of SSRs were identified, supplying the basis for the development of molecular markers. Moreover, a complete mitochondrial genome was assembled from the sequencing data. Potential SNPs were screened out by comparing mitochondrial gene sequences obtained in this study with the published mitochondrial genome of *Provanna* sp. from a methane seep in the South China Sea. In addition to *cox1*, it was proposed that *nad2*, *nad4*, *nad5*, and *cob* can be employed as markers for population genetics and phylogenetic analysis of *P. glabra* and its relatives. Non-synonymous mutations predominantly occur in *nad* genes, which may play an important role in *Provanna* snails’ adaptation to different environments. The phylogenetic tree based on mitochondrial genomes established the evolutionary position of *P. glabra*, and rapid evolutionary rates were detected in three mitochondrial genes of deep-sea species, suggesting that mitochondrion experienced stronger selection compared with shallow-water gastropods. These results provide genome resources, evidence, and candidate molecular markers for the studies of adaptive evolution in snails from deep-sea chemosynthetic ecosystems and lay a foundation for the subsequent high-quality genome map construction of *P. glabra*.

## Figures and Tables

**Table 1:** Statistics on the genome survey sequencing data and assembled draft genome of *P. glabra*.

**Sequencing Data**	
Raw bases (Gb)	63.54
Q30 of raw data (%)	88.50
GC content of raw data (%)	45.13
Clean bases (Gb)	55.70
Q30 of clean data (%)	92.98
GC content of clean data (%)	44.48
**Genome Assembly**	
Number of contigs	4,132,680
Contig N50 (bp)	437
Number of scaffolds	3,534,913
Scaffold N50 (bp)	581
GC content of the assembly (%)	45.52

**Table 2:** Statistics on transposal elements (TEs) in the assembled draft genome of *P. glabra*.

	RepBase		TE Proteins		De Novo		Combined TEs	
	Length (bp)	Percentage in Genome	Length (bp)	Percentage in Genome	Length (bp)	Percentage in Genome	Length (bp)	Percentage in Genome
**DNA**	91,183,057	6.88%	4,798,383	0.36%	35,706,323	2.70%	124,415,616	9.39%
**LINE**	31,192,761	2.35%	30,457,352	2.30%	47,851,776	3.61%	81,713,306	6.17%
**SINE**	7,573,630	0.57%	-	-	57,325,068	4.33%	59,987,677	4.53%
**LTR**	27,154,235	2.05%	3,528,767	0.27%	21,823,849	1.65%	49,670,310	3.75%
**Other**	33,747	-	1401	-	-	-	35,148	-
**Unknown**	3,016,383	0.23%	15,420	-	243,550,406	18.38%	246,409,803	18.60%
**Total**	139,568,993	10.53%	38,782,100	2.93%	401,595,868	30.31%	532,071,289	40.17%

**Table 3:** Information of putative SNPs within the mitochondrial genome of *P. glabra*.

Gene Name	Gene Length (bp)	Mutation Number (Percentage)	Amino Acid Change	Variation Site (DNA)	Mutation Type
*cox1*	1536	33 (2.15%)	-	-	-
*cox2*	687	20 (2.91%)	-	-	-
*cox3*	780	29 (3.72%)	A→G	527	Transversion
			T→A	592	Transition
*nad1*	942	19 (2.02%)	-	-	-
*nad2*	1062	32 (3.01%)	S→N	242	Transition
				243	
			V→I	502	
			A→I	886	
				887	
			M→V	961	
*nad3*	354	13 (3.67%)	I→T	263	Transition
			S→F	281	
			T→M	284	
*nad4*	1362	43 (3.16%)	D→N	544	Transition
*nad4l*	258	7 (2.71%)	S→T	280	Transversion
*nad5*	1722	62 (3.60%)	I→T	512	Transition
			Y→C	1196	
			I→V	1402	
			D→N	1432	
			V→I	1699	
*nad6*	501	16 (3.19%)	S→P	244	Transition
*cob*	1140	42 (3.68%)	V→A	704	Transition
*atp6*	696	12 (1.72%)	-	-	-
*atp8*	159	3 (1.89%)	-	-	-

## Data Availability

The data supporting the conclusions of this article will be made available by the authors without undue reservation. The clean data used in the genome survey are already publicly available in the NCBI SRA database with the accession number PRJNA990319. The mitochondrial genome sequence of the snail is deposited in GenBank under the accession number OR209184.

## Supplementary Materials

The following supporting information can be downloaded at: https://www.mdpi.com/article/10.3390/ani13213313/s1, Table S1: Information on species included in the phylogenetic analysis; Table S2: BUSCO evaluation of the draft genome of *P. glabra*; Table S3: Statistics of repeat sequences in the draft genome of *P. glabra*; Table S4: Proportions of TE types in different species; Table S5: Statistics of SSRs in the draft genome of *P. glabra*; Table S6: Information on mitochondrial genome of *P. glabra*; Table S7: Evolutionary rate comparison of mitochondrial genes in the deep-sea and shallow-water gastropod lineages.
