# Supplementary material for: The First Genome Survey of the Snail Provanna glabra Inhabiting Deep-Sea Hydrothermal Vents
Source: Animals (Basel). 2023 Oct 25;13(21):3313. doi: 10.3390/ani13213313 (PMC10648102; doi:10.3390/ani13213313)
Supplement: Supplementary file 1 [file animals-13-03313-s001.zip › animals-2612936-supplementary.pdf]

Table S1. Information of species included in the phylogenetic analysis.

Species	Accession No.
Amphioctopus marginatus (outgroup)	NC_036351
Alviniconcha marisindica	OQ695489
Bolinus brandaris	NC_013250
Conus borgesi	NC_013243
Dendropoma maximum	NC_014583
Ifremeria nautilei	KC757644
Monoplex parthenopeus	NC_013247
Oncomelania hupensis	NC_013073
Provanna.sp	KM675481
Haliotis rubra	NC_005940
Albinaria caerulea	NC_001761
Aplysia californica	NC_005827
Pupa strigosa	NC_002176
Onchidella celtica	NC_012376
Pyramidella dolabrata	NC_012435
Roboastra europaea	NC_004321
Provanna glabra (this study)	OR209184
Gigantopelta aegis	MW442948
Pomacea canaliculata	NC_024586
Lottia digitalis	NC_007782
Alviniconcha adamantis	NC_080997
Nerita melanotragus	GU810158

Table S2. BUSCO evaluation for the draft genome of *P. glabra*.

C	D	F	M
68 (7.00%)	5 (0.50%)	439 (44.90%)	466 (47.60%)

C, complete and single-copy BUSCOs. D, complete and duplicated BUSCOs. F, fragmented BUSCOs. M, missing BUSCOs.

Table S3. Statistics of repeat sequences in the draft genome of *P. glabra*.

	Repeat Size(bp)	Percentage of genome(%)
**TRF**	173,899,956	13.13
**Repeatmasker**	147,187,607	11.11
**RepeatProteinMask**	38,782,100	2.93
De novo	403,602,007	30.46
**Total**	636,277,514	48.03

Table S4. Proportions of TE types in different species.

Species		TEs type				References
		DNA transposons	LTRs	LINEs	SINE	
Provanna glabra	deep-sea	9.39%	3.75%	6.17%	4.53%	
Chrysomallon squamiferum	deep-sea	17.73%	5.99%	5.65%	*	
Gigantopelta aegis	deep-sea	32.15%	13.32%	11.51%	*	[28]
Bathyacmaea lactea	deep-sea	10.4%	0.7%	6.5%	5.5%	[33]
Pomacea canaliculate	shallow water	6.84%	3.53%	8.63%	*	
Biomphalaria glabrata	shallow water	20.20%	3.75%	23.93%	*	[28]
Batillaria attramentaria	shallow water	3.81%	*	4.83%	5.55%	[34]
Elysia chlorotica	shallow water	6.2%	2.7%	5.6%	3.6%	[35]

Note: “*” represents a very low proportion of the TE type or not described in the original articles.

Table S5. Statistics of SSRs in the draft genome of *P. glabra*.

SSR type	Mono-nucleotide	Di-nucleotide	Tri-nucleotides	Tetra-nucleotides	Penta-nucleotides	Hexa-nucleotides	Total
**Number**	286,496	1,265,214	526,178	260,080	42,217	6,985	2,387,170
**Percentage**	12.00%	53.00%	22.04%	10.90%	1.77%	0.29%	100

Table S6. Information of mitochondrial genome of *P. glabra.*

Gene	Positions			Codons		
	Start	Stop	Length(bp)	Start/Stop	overlap	Strand
cox1	1	1536	1536	ATG/TAA	22	+
cox2	1559	2245	687	ATG/TAA	8	+
tRNA-Asp (GUC)	2254	2321	68		0	+
atp8	2322	2480	159	ATG/TAA	2	+
atp6	2483	3178	696	ATG/TAA	34	+
tRNA-Met (CAU)	3213	3280	68		1	-
tRNA-Tyr (GUA)	3282	3353	72		1	-
tRNA-Cys (GCA)	3355	3421	67		0	-
tRNA-Trp (UCA)	3422	3487	66		-3	-
tRNA-Gln (UUG)	3485	3551	67		6	-
tRNA-Gly (UCC)	3558	3624	67		0	-
tRNA-Glu (UUC)	3625	3695	71		0	-
rrnS (12S rRNA)	3696	4650	955		-5	+
tRNA-Val (UAC)	4646	4712	67		-18	+
rrnL (16S rRNA)	4695	6109	1415		2	+
tRNA-Leu2 (UAA)	6112	6180	69		35	+
tRNA-Leu1 (UAG)	6216	6284	69		-1	+

nad1	6284	7225	942	ATG/TAA	0	+
tRNA-Pro (UGG)	7226	7292	67		3	+
nad6	7296	7796	501	ATG/TAA	8	+
cob	7805	8944	1140	ATG/TAA	13	+
tRNA-Ser2 (UGA)	8958	9205	248		-180	+
tRNA-Thr (UGU)	9026	9093	68		13	-
nad4l	9107	9403	297	ATG/TAG	5	+
nad4	9409	10770	1362	ATC/TAA	3	+
tRNA-His (GUG)	10774	10841	68		-1	+
nad5	10841	12562	1722	ATG/TAA	3	+
tRNA-Phe (GAA)	12566	12633	68		0	+
cox3	13577	14356	780		41	+
tRNA-Lys (UUU)	14398	14468	71		9	+
tRNA-Ala (UGC)	14478	14547	70		7	+
tRNA-Arg (UCG)	14555	14623	69		6	+
tRNA-Asn (GUU)	14630	14698	69		7	+
tRNA-Ile (GAU)	14706	14775	70		4	+
nad3	14780	15133	354	ATG/TAA	1	+
tRNA-Ser1 (GCU)	15135	15202	68		0	+
nad2	15203	16264	1062	ATG/TAA	3	+

Table S7. Evolutionary rate comparison of mitochondrial genes in the deep-sea and shallow water gastropod lineages.

Gene name	Model 0	Model 2		LRT *P*-value
	ω	ω0	ω1	
cox1	0.00362	0.00215	0.00522	**0.00597**
cox2	0.01198	0.00901	0.0152	0.13296
cox3	0.01305	0.00667	0.01815	**0.00278**
cob	0.01444	0.01589	0.01375	0.58174
atp6	0.01714	0.01234	0.02033	0.11447
atp8	0.03976	0.05319	0.03453	0.58174
nad1	0.01346	0.0145	0.01312	0.79954
nad2	0.01714	0.01234	0.02033	0.11447
nad3	0.02985	0.0086	0.03475	0.17008
nad4	0.02589	0.00135	0.02897	**0.00333**
nad4l	0.01126	0.03953	0.00721	**0.00721**
nad5	0.02525	0.03201	0.02293	0.12336
nad6	0.03571	0.03062	0.038	0.57723

Note: In Model 0, the ω value represents the overall dN/dS ratio across all branches; in Model 2, ω0 represents dN/dS ratio of shallow water species branch (background lineage), and ω2 represents deep-sea species branch (foreground lineage). *P*-values<0.01 in bold means significant difference between M0 and M2 at this gene.
